# Digital Engagement Significantly Enhances Weight Loss Outcomes in Adults With Obesity Treated With Tirzepatide: Retrospective Cohort Study of a Digital Weight Loss Service

**DOI:** 10.2196/83718

**Published:** 2026-01-15

**Authors:** Hans Johnson, Ashley Kieran Clift, Daniel Reisel, David Huang

**Affiliations:** 1 Engineering and Physical Sciences Research Council Centre for Doctorate Training in Digital Health and Care University of Bristol Bristol United Kingdom; 2 Department of Clinical Innovation and Research Voy (T/a Menwell LTD) London United Kingdom; 3 Department of Education University of Oxford Oxford United Kingdom; 4 Department of Surgery and Cancer Imperial College London London United Kingdom; 5 Elizabeth Garrett Anderson Institute for Women's Health University College London London United Kingdom

**Keywords:** obesity, weight loss, tirzepatide, digital health, engagement, behavior, coaching, retrospective study, GIP/GLP-1 RA, glucose-dependent insulinotropic polypeptide and glucagon-like peptide-1 receptor agonist

## Abstract

**Background:**

The advent of tirzepatide has transformed obesity care; yet, real-world weight loss outcomes necessarily depend on patient engagement with behavioral support. Digital platforms offering coaching, self-monitoring, and automated feedback have the potential to further augment pharmacological efficacy.

**Objective:**

The aim of the study is to examine associations between digital engagement and weight loss outcomes among adults prescribed tirzepatide in routine care over 12 months and to identify baseline correlates of engagement.

**Methods:**

In this retrospective cohort study, we included adults (18-75 years; BMI ≥30 or ≥27.5 kg/m^2^ with comorbidities) who initiated tirzepatide between February 2024 and August 2025 via a UK digital weight loss service. Engagement was defined by all 3: attendance at ≥1 coaching session AND ≥1 weekly weight log AND ≥1 app login over 12 months. Percent weight loss was analyzed at months 2, 4, 6, 8, 10, and 12 using a mixed model repeated measures adjusted for age, sex, baseline BMI, and comorbidities. Time-to-event analyses (Kaplan-Meier) assessed attainment of ≥5%, ≥10%, ≥15%, and ≥20% weight loss thresholds. Multivariable logistic regression identified correlates of engagement, reporting odds ratios (ORs) per decade of age and per 5 kg/m^2^ BMI.

**Results:**

Among 126,553 participants, 6746 (5.3%) were maximally engaged. Cohort demographics were a mean age of 42.3 (SD 12.4) years, 78.9% (99,905/126,553) female, and a mean BMI of 35.3 (SD 6.2) kg/m^2^. Engaged users achieved greater adjusted weight loss at month 12 (–22.9%, 95% CI –23.2 to –22.6) versus nonengaged users (–17.5%, 95% CI –17.7 to –17.4), an absolute difference of 5.3 percentage points (*P<*.001; Cohen *d*=0.54). Differences emerged by month 2 (–7.4% vs –6.4%; *P<*.001) and widened steadily. Engaged participants reached all clinically significant weight loss thresholds faster (5%-20%; log-rank *P<*.001), and engaged participants were nearly 3 times more likely to achieve ≥20% weight loss compared to nonengaged participants (1079/6746, 16% vs 6710/119,807, 5.6%; risk ratio 2.88; *P<*.001). Older age (OR 1.18 per decade, 95% CI 1.15-1.20; *P<*.001), higher BMI (OR 1.14 per 5 kg/m^2^, 95% CI 1.12-1.16; *P<*.001), and the presence of polycystic ovary syndrome (OR 1.59, 95% CI 1.45-1.74; *P<*.001) or fatty liver disease (OR 1.52, 95% CI 1.32-1.76; *P<*.001) correlated with engagement. Male sex (OR 0.86, 95% CI 0.81-0.92; *P<*.001) and diabetes (OR 0.83, 95% CI 0.73-0.95; *P*=.009) were associated with lower engagement.

**Conclusions:**

Digital engagement was associated with substantially greater tirzepatide-associated weight loss in real-world practice. Integrating structured digital support with pharmacotherapy represents a promising strategy for optimizing obesity management.

## Introduction

### Background

The global prevalence of obesity has tripled since 1975, and the economic burden of obesity and overweight is projected to reach 3.3% of global gross domestic product by 2060 [1]. Beyond economic considerations, obesity substantially increases risks for numerous chronic conditions including type 2 diabetes, cardiovascular disease, certain cancers, as well as overall mortality. Despite recognition of obesity as a complex chronic disease requiring multifaceted interventions, historically available treatments have shown limited long-term efficacy [2].

Recent advancements in pharmacotherapy have transformed the obesity treatment paradigm. Tirzepatide (Mounjaro) is a novel dual glucose-dependent insulinotropic polypeptide (GIP) and glucagon-like peptide-1 (GLP-1) receptor agonist (RA) that has demonstrated remarkable efficacy for weight management in randomized controlled trials (RCTs) [3]. The SURMOUNT-1 trial reported mean weight reductions of 15% to 20.9% at 72 weeks, significantly surpassing previous pharmacotherapies [4]. These results have generated substantial clinical interest; yet, questions remain regarding their generalization to real-world settings and the scope for adjuncts to support or optimize outcomes.

Digital weight loss services (DWLSs) combine pharmacotherapy with app-based tools, regular weight tracking, and professional health education and tailor nutrition-driven coaching. These services may address critical barriers to obesity treatment, including limited health care provider time, accessibility challenges, and inadequate behavioral support [5-7]. Understanding how digital engagement impacts pharmacotherapy outcomes could critically inform clinical recommendations and health service design. Furthermore, digital health interventions represent a promising complement to pharmacotherapy. Recent systematic reviews indicate that technology-based approaches can enhance weight loss outcomes by improving self-monitoring, accountability, personalized feedback, and remote support [8-10]. Studies of remotely delivered weight loss services have shown the efficacy of such digital intervention [11]. However, apart from a few recent studies [5], there is limited evidence regarding how digital engagement influences outcomes specifically in patients using dual GLP-1 and GIP RA for weight management.

### Objectives

This study conducted a retrospective evaluation of the Voy DWLS, which is available throughout the United Kingdom. Our objectives were to characterize the real-world effectiveness of tirzepatide on weight loss, explore the associations between digital engagement and outcomes, and identify factors that correlate with engagement.

## Methods

### Study Design and Setting

This retrospective study used an open cohort approach. The study period spanned early February 2024 to early August 2025, with patients contributing follow-up from the date of first prescription to the earliest date of last weight measurement logged or reached 12 months of follow-up. The Voy digital health platform, a commercial telehealth DWLS for obesity management, was developed by a multidisciplinary team of clinicians, behavioral scientists, and software developers to provide remote behavioral support through live group video coaching sessions, text-based in-app support, dynamic educational content, and the direct supply of tirzepatide for weight management. Drawing upon established frameworks in behavior change and self-management support, the platform combined core digital tools (eg, real-time weight monitoring, medication adherence tracking, and personalized coaching sessions) into a single interface accessible via smartphone or web browser.

### Procedure

**Figure 1:**
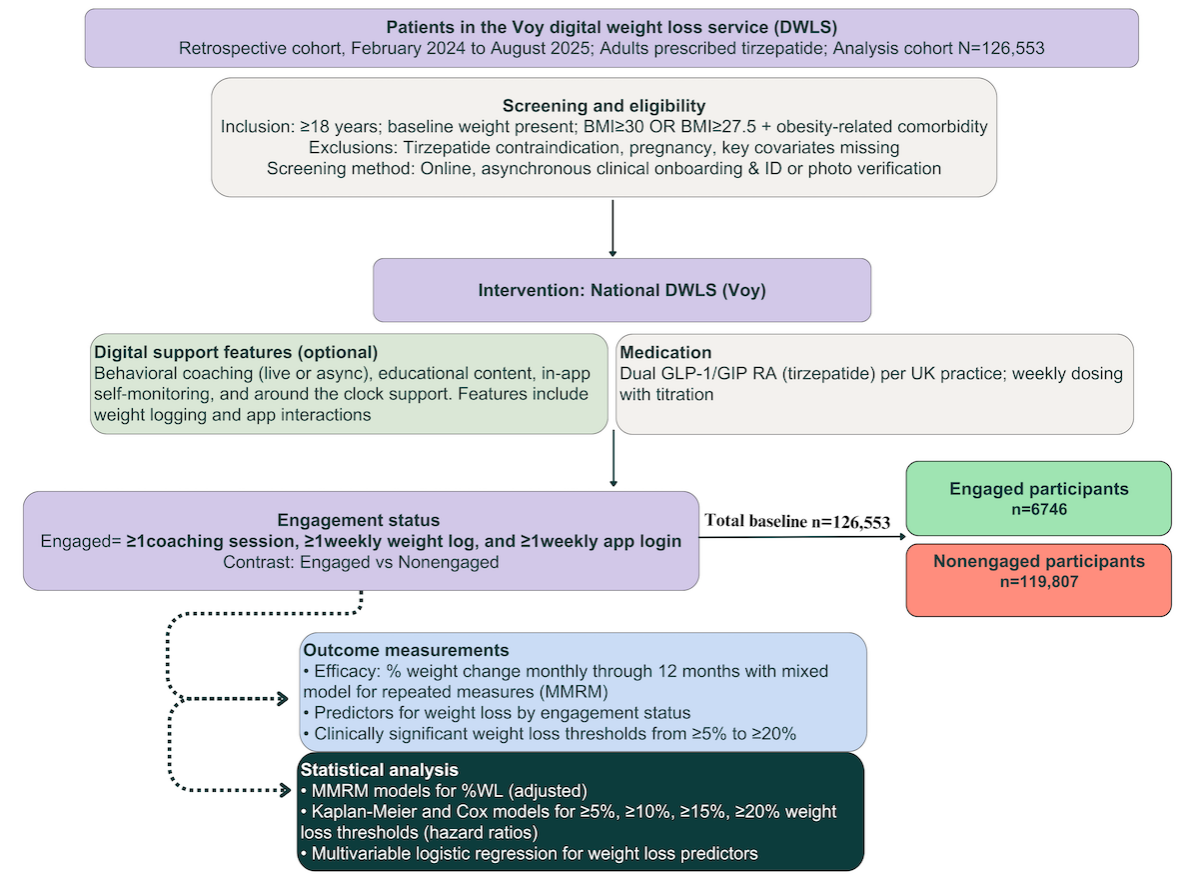
Flowchart illustrating the critical steps of the cohort retrospective analysis conducted for the Voy digital weight management program. GIP: glucose-dependent insulinotropic polypeptide; GLP-1: glucagon-like peptide-1; RA: receptor agonist.

Participants became aware of the Voy program through multiple channels: targeted social media campaigns, clinician referrals, word-of-mouth recommendations, and general web searches. They self-enrolled and self-paid via the Voy website, where they completed an online screening questionnaire covering medical history, BMI, and lifestyle factors. Thereafter, the participants interacted with the Voy digital health platform’s weight management program, which integrates dual GLP-1 and GIP RA pharmacotherapies with digital behavioral support to enhance weight loss outcomes. Subsequently, participants self-enrolled via the Voy website, where they completed an online screening questionnaire covering medical history, BMI, and a range of lifestyle factors. Upon enrollment, participants underwent an initial assessment to confirm eligibility, including verification of age, BMI, and the absence of exclusion criteria (Figure 1). All participants underwent comprehensive clinical screening before treatment initiation. Structured safeguards ensured accuracy and safe prescribing: photo identification and full-body photographs for identity and BMI verification; detailed clinical questionnaires capturing medical history, contraindications, and current medications; and individual review by qualified licensed clinical prescribers with cross-checking for clinical red flags. The service operates under Care Quality Commission registration with internal clinical audit processes, escalation protocols for safety concerns, and regular safety reviews. This approach adheres to General Medical Council and General Pharmaceutical Council standards for remote prescribing in the United Kingdom, with clinician-led prescribing decisions and independent verification of eligibility criteria to ensure patient safety.

The initial monthly cost of enrollment for tirzepatide was US $273.72. Participants receive tirzepatide (Mounjaro) via multidose prefilled KwikPen containing 4 weekly doses. Administration is a subcutaneous injection once weekly, with doses ranging from 2.5 to 15 mg. The starting dose is 2.5 mg, increased every 4 weeks in 2.5 mg increments as tolerability permits. New tirzepatide pens are prescribed every 28 days. Monthly costs cover medication, clinical support from qualified prescribing clinicians, personalized coaching from registered dietitians and nutritionists, and access to digital tracking tools and educational content (see Multimedia Appendix 1 for detailed program description). Although online weight tracking was mandatory for continued prescriptions, every other component of the Voy platform was optional.

Participants attended group onboarding sessions and were offered fortnightly coaching to enhance engagement and adherence. Coaches were trained based on principles from social cognitive theory, self-determination theory, the transtheoretical model, and the theory of planned behavior [12,13]. These techniques focused on fostering intrinsic motivation, goal setting, and problem-solving to promote sustainable lifestyle changes tailored to participants’ individual progress.

### Participants

Participants were adult residents of the United Kingdom, aged between 18 and 75 years, with a BMI of 30 kg/m^2^ or higher or >27.5 kg/m^2^ with obesity-related comorbidities who were enrolled into the Voy digital weight loss service and were prescribed tirzepatide.

### Eligibility Criteria

Eligibility required access to a smartphone or tablet. Exclusion criteria included history of self-reported eating disorders (eg, anorexia nervosa and bulimia nervosa), pregnancy or patients seeking fertility, known allergies or hypersensitivity to any components of tirzepatide, and severe medical conditions with established contraindications for tirzepatide as per Multimedia Appendix 2.

### Data Completeness

Engagement metrics and weight loss outcomes were available for the entire sample of participants who used the digital platform during the study period at baseline. Complete demographic information (eg, age, sex, and comorbidities) was available for the entire sample participants who enrolled during the initial phase of the service.

### Defining Engagement and Outcome

#### Primary Outcome

The primary outcome was percentage weight loss from baseline. Weight measurements were entered directly in the app by participants.

#### Secondary Outcomes of Interest Included: Digital Engagement as an Exposure Variable

Engagement was defined based on a patient engaging in all 3 key behaviors identified as a priority by the Voy clinical and research teams as likely associated with weight loss outcomes. These behaviors are as follows:

- Coaching session attendance: participation in group or individual coaching sessions (video, audio, or text-based).
- Weight-tracking frequency: regular logging of body weight in the app.
- App use and logins: logging into the platform to view educational content, track health metrics, or interact with coaches.

As in our prior work [5], participants were classified as “engaged” if they met all of the following criteria: attended ≥1 coaching session AND tracked weight ≥1 per week AND logged into the app at least once during the study period. Those who did not fulfill all 3 of these criteria as a compound were classified as “nonengaged.”

### Statistical Analysis

#### Longitudinal Weight Loss Analysis

Our primary analysis used a mixed model for repeated measures (MMRM) approach, informed by the statistical methodology of the SURMOUNT-1 RCT study [4]. This approach accounted for repeated weight measurements for individuals, varying follow-up duration, and implicitly handled missing data without imputation under the missing at random assumption. The model incorporated fixed effects for digital engagement status, months, and the interaction between engagement and month. To adjust for potential confounding variables, we included sex, age, baseline BMI, and comorbid conditions as covariates (see Multimedia Appendix 3 for detailed methodology).

#### Sensitivity Analyses for Follow-Up Duration

To address the rolling enrollment design, we conducted sensitivity analyses restricted to subcohorts reaching specific follow-up milestones (3, 6, 9, and 12 months). Linear regression models were fitted for each subcohort with percentage weight change from baseline as the dependent variable, adjusted for the same confounders as the MMRM analyses. Marginal estimates (least squares means) were calculated from these models, with contrast *P* values comparing engaged versus nonengaged groups. These analyses assessed whether associations between engagement and weight loss remained consistent across different follow-up durations.

#### Postmodel Analyses and Effect Size Calculation

For postmodel analyses, we calculated adjusted means and conducted pairwise comparisons between engagement groups using the *emmeans* package. Significance testing of the engagement effect at each time point used 2-sided tests with α=.05, without adjustment for multiple comparisons, consistent with standard approaches for longitudinal repeated measures designs.

To quantify the magnitude and clinical relevance of the engagement effect, we calculated effect sizes using multiple approaches. Cohen *d* values were computed using the estimated difference divided by the pooled SD. Additionally, we computed both absolute differences (in percentage points) and relative differences (as a percentage of the nonengaged group’s weight loss) between engaged and nonengaged tirzepatide users. To examine whether the impact of engagement on weight loss varied over time, we conducted a formal test of the engagement by time interaction.

#### Data Processing and Descriptive Statistics

All data processing and analyses were conducted using R software (version 4.3.1; R Foundation for Statistical Computing). Baseline characteristics were summarized using means and SDs for continuous variables, while frequencies and percentages were calculated for categorical variables. These statistics were calculated separately for engaged and nonengaged groups.

#### Baseline Correlates of Digital Engagement

Baseline characteristics associated with digital engagement were identified using multivariable logistic regression, where the dependent variable was digital engagement status (engaged vs not engaged, defined as mentioned earlier). Independent variables included demographic characteristics (age and sex), baseline BMI, and documented comorbidities (diabetes, hypertension, hypercholesterolemia, polycystic ovary syndrome [PCOS], and fatty liver disease). Age was modeled as a continuous variable, with additional transformations to express odds ratios (ORs) per decade increase. Similarly, BMI was analyzed with secondary calculations to express ORs per 5 kg/m^2^ increase.

#### Achievement of Clinically Significant Weight Loss

Kaplan-Meier methods were used to estimate the proportions of users attaining ≥5%, ≥10%, ≥15%, and ≥20% weight loss during the study period with 95% CIs. This was performed for the cohort overall, then comparing engaged and nonengaged groups. Log-rank tests were used to compare cumulative incidence curves. Hazard ratios (HRs) with 95% CIs were calculated to quantify the relative rate of achieving weight loss thresholds between engagement groups, and risk ratios (RRs) were computed to assess the relative probability of threshold achievement.

#### Sample Size

A power analysis determined that a minimum of 118 participants per engagement group would provide 80% power to detect a 15% difference in the proportion of participants achieving ≥10% weight loss at a 5% significance level.

### Bias and Missing Data

Self-reported weight measurements could introduce reporting bias. To mitigate this, participants were encouraged to provide accurate measurements through regular reminders and had the option to upload progress photographs, enhancing data validity. Additionally, data validation checks were performed internally and by statisticians to identify and address implausible values. Selection bias was minimized by including all eligible participants who initiated the program within the study period, ensuring the sample was representative of the population using the DWLS.

Missing data were addressed through the MMRM approach, which uses all available observations without requiring complete cases. This method operates under the missing at random assumption, whereby missingness may depend on observed covariates and previous measurements but not on unobserved values conditional on observed data. MMRM provides unbiased parameter estimates and maintains statistical efficiency in the presence of intermittent missing data, consistent with statistical methodology for landmark obesity trials [4]. For time-to-event analyses, participants who remained on treatment were censored at their last prescription date or study end, with censoring appropriately incorporated into Kaplan-Meier and Cox regression models.

### Ethical Considerations

This retrospective open cohort study was an analysis of deidentified data collected during the routine clinical care of adults treated by Voy. The study was approved by the University College London Research Ethics Committee (Project ID 2025-0906-775). The study adhered to the principles outlined in the Declaration of Helsinki. Participants provided informed consent for their anonymized data to be used for ethically approved research and service improvement purposes upon enrollment in the program. To protect participant privacy, all data were deidentified prior to analysis, with direct identifiers (including names, contact details, and unique patient identifiers) removed and replaced with study-specific codes. Data were stored on secure servers with access restricted to authorized research personnel only. Participants did not receive any compensation for their participation in this study, as the analysis was conducted retrospectively using data collected during routine clinical care.

### Adherence to STROBE Guidelines

This study adhered to the STROBE (Strengthening the Reporting of Observational Studies in Epidemiology) guidelines [14] (Multimedia Appendix 4).

## Results

### Participant Characteristics

Among 126,553 participants who initiated tirzepatide, 6746 (5.3%) met criteria for maximally digital engagement. Baseline characteristics stratified by engagement status are presented in Table 1. The overall cohort had a mean age of 42.3 (SD 12.4) years, was predominantly female (99,905/126,553, 78.9%), and had a mean baseline BMI of 35.3 (SD 6.2) kg/m^2^. Compared to nonengaged participants, digitally engaged individuals were significantly older (engaged: mean 44.9, SD 12.0 years vs nonengaged: mean 42.2, SD 12.5 years; *P<*.001), more likely to be female (engaged: 5486/6746, 81.3% vs nonengaged: 94,419/119,807, 78.8%; *P<*.001), and had higher baseline BMI (engaged: mean 36.4, SD 6.7 kg/m^2^ vs nonengaged: mean 35.2, SD 6.2 kg/m^2^; *P<*.001). Engaged participants also reported higher prevalence of comorbidities, including type 2 diabetes (engaged: 250/6746, 3.7% vs nonengaged: 3681/119,807, 3.1%; *P<*.001), hypertension (engaged: 978/6746, 14.5% vs nonengaged: 11,592/119,807, 9.7%; *P<*.001), hypercholesterolemia (engaged: 707/6746, 10.5% vs nonengaged: 7738/119,807, 6.5%; *P<*.001), and PCOS (engaged: 591/6746, 8.8% vs nonengaged: 7285/119,807, 6.1% of female participants; *P<*.001).

**Table 1:** Baseline demographic and clinical characteristics of participants by digital engagement status.

Characteristic		Overall (N=126,553)	Digitally engaged (n=6746)	Not digitally engaged (n=119,807)	*P* value^a^	
**Age (years)**						<.001
	Mean (SD)	42.3 (12.4)	44.9 (12.0)	42.2 (12.5)		
**Age group (years), n (%)**						<.001
	18-24	6892 (5.4)	120 (1.8)	6772 (5.7)		
	25-34	32,037 (25.3)	1399 (20.7)	30,638 (25.6)		
	35-44	36,334 (28.7)	1977 (29.3)	34,357 (28.7)		
	45-54	27,269 (21.5)	1643 (24.4)	25,626 (21.4)		
	55+	24,016 (19)	1607 (23.8)	22,409 (18.7)		
**Sex, n (%)**						<.001
	Female	99,905 (78.9)	5486 (81.3)	94,419 (78.8)		
	Male	26,648 (21.1)	1260 (18.7)	25,388 (21.2)		
**Anthropometric measures, mean (SD)**						
	Weight (kg)	98.3 (20.4)	101.9 (21.5)	98.1 (20.4)	<.001	
	Height (cm)	166.7 (9.1)	167.1 (8.7)	166.7 (9.2)	.003	
	BMI (kg/m^2^)	35.3 (6.2)	36.4 (6.7)	35.2 (6.2)	<.001	
**BMI category, n (%)**						<.001
	Overweight (25-29.9 kg/m^2^)	13,084 (10.4)	841 (12.5)	12,243 (10.2)		
	Obese (≥30 kg/m^2^)	113,246 (89.6)	5871 (87.5)	107,375 (89.8)		
**Comorbidities, n (%)**						
	Diabetes mellitus	3931 (3.1)	250 (3.7)	3681 (3.1)	.009	
	Hypertension	12,570 (9.9)	978 (14.5)	11,592 (9.7)	<.001	
	Hypercholesterolemia	8445 (6.7)	707 (10.5)	7738 (6.5)	<.001	
	PCOS^b^	7876 (6.2)^c^	591 (10.8)^c^	7285 (6.1)^c^	<.001	
	Nonalcoholic fatty liver	2391 (1.9)	229 (3.4)	2162 (1.8)	<.001	
**Digital engagement metrics, n (%)**						
	Coaching sessions attended	37,224 (29.4)	6746 (100)	30,478 (25.4)	<.001	
	Weight tracking ≥1 per week readings	18,992 (15)	6746 (100)	12,246 (10.2)	<.001	
	Uses weight loss app^d^	93,627 (74)	6746 (100)	86,881 (72.5)	<.001	

^a^*P* values calculated using independent 2-tailed *t* tests for continuous variables and chi-square tests for categorical variables.

^b^PCOS: polycystic ovary syndrome.

^c^Percentage calculated among female participants only.

^d^Defined as having logged into the app at least once during enrollment and used app features with the exception of weight measurement.

### Engagement Characteristics

Among engaged participants (n=6746), all met the 3 required criteria by definition: coaching session attendance (mean 1.00, SD 0.00), app use (mean 1.00, SD 0.00), and weekly weight tracking (mean 1.00, SD 0.00). Among nonengaged participants (n=119,807), partial engagement was common: 86,881 (72.5%) used the app (mean 0.73, SD 0.45), 18,864 (15.7%) attended coaching (mean 0.16, SD 0.36), and tracking adherence (mean 0.09, SD 0.27) was lower. These patterns indicate that while most participants used some platform features, especially 93,627 of 126,553 (74%) of the overall cohort using the digital app, simultaneous engagement across all 3 modalities was achieved by 6,746 of 126,553 (5.3%) of users.

### Weight Loss Outcomes

#### Overview

**Figure 2:**
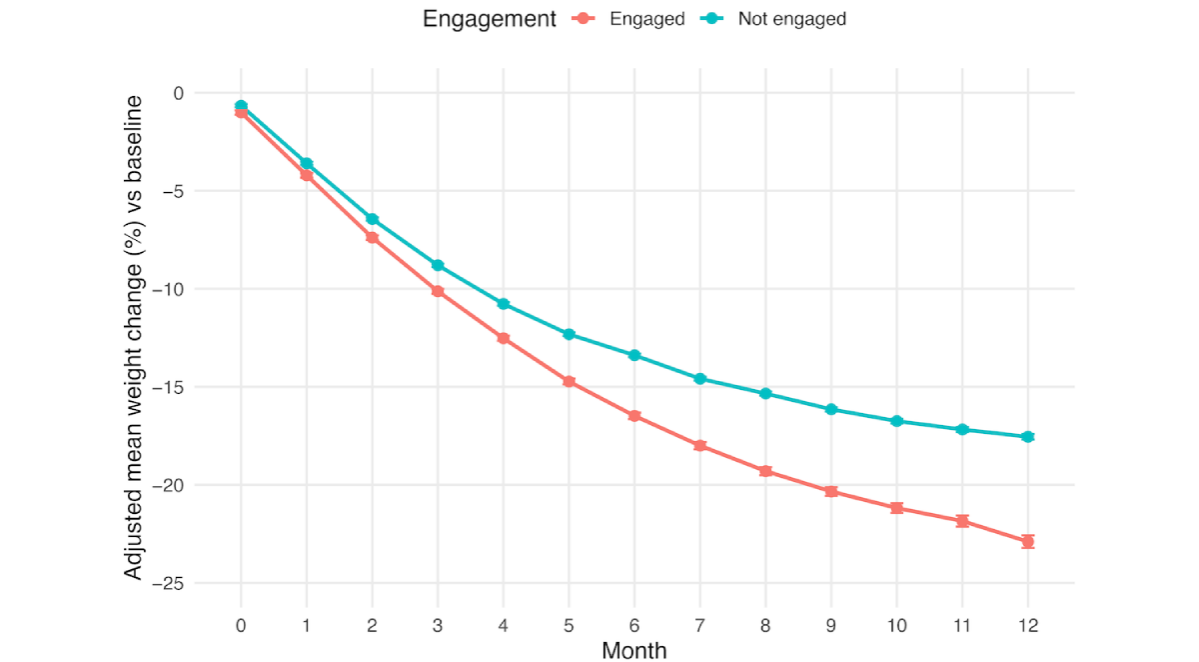
Mean adjusted mixed model repeated measure derived and confounder-adjusted percentage weight loss from baseline among tirzepatide users stratified by digital engagement status.

Figure 2 displays the MMRM model with compound symmetry covariance adjusted for confounders, representing percentage weight loss over 12 months by engagement status. Both digitally engaged and nonengaged groups demonstrated progressive weight loss throughout the observation period, but differences between groups emerged early and increased over time. Table 2 presents detailed weight loss outcomes at each time point.

**Table 2:** Percentage weight loss by digital engagement status in participants taking tirzepatide (months 0-12), estimated using mixed model repeated measure (MMRM).

Month and engagement status			Participants, n		Adjusted mean % weight loss^a^ (95% CI)		Absolute difference^b^ (percentage points)		Relative difference^c^ (%)		Effect size (Cohen *d*)		*P* value^d^
**Month 0**													
	Not engaged	119,807		0		—^e^		—		—		—	
	Engaged	6746		0		—		—		—		—	
**Month 2**													
	Not engaged	40,582		–6.44 (–6.53 to –6.36)		0.95		14.8		0.083		<.001	
	Engaged	5751		–7.39 (–7.51 to –7.27)		—		—		—		—	
**Month 4**													
	Not engaged	21,762		–10.77 (–10.86 to –10.68)		1.75		16.2		0.207		<.001	
	Engaged	3420		–12.52 (–12.65 to –12.39)		—		—		—		—	
**Month 6**													
	Not engaged	13,559		–13.39 (–13.48 to –13.30)		3.09		23.1		0.422		<.001	
	Engaged	1724		–16.48 (–16.64 to –16.32)		—		—		—		—	
**Month 8**													
	Not engaged	6704		–15.34 (–15.45 to –15.24)		3.96		25.8		0.464		<.001	
	Engaged	896		–19.30 (–19.51 to –19.09)		—		—		—		—	
**Month 10**													
	Not engaged	3918		–16.75 (–16.87 to –16.63)		4.43		26.4		0.467		<.001	
	Engaged	567		–21.18 (–21.42 to –20.93)		—		—		—		—	
**Month 12**													
	Not engaged	2343		–17.55 (–17.69 to –17.41)		5.34		30.4		0.539		<.001	
	Engaged	310		–22.89 (–23.22 to –22.57)		—		—		—		—	

^a^Adjusted means derived from MMRM with compound symmetry covariance structure, controlling for age, sex, baseline BMI, diabetes, hypertension, high cholesterol, polycystic ovary syndrome, and fatty liver disease.

^b^Absolute difference=engaged group mean–not engaged group mean (percentage points).

^c^Relative difference=(absolute difference/not engaged group mean)×100%.

^d^*P* values from the model time×engagement interaction terms.

^e^Not applicable.

#### Weight Loss by Engagement Status

By month 12, after adjusting for potential confounders (age, sex, baseline BMI, and comorbidities) in MMRM analysis, digitally engaged participants achieved a mean weight loss of 22.9% (95% CI 22.6-23.2) compared to 17.6% (95% CI 17.4-17.7) among nonengaged participants, representing an absolute difference of 5.3 percentage points (*P<*.001). The effect size (Cohen *d*=0.539) indicated a medium clinical effect of digital engagement on weight loss outcomes.

The divergence in adjusted weight loss trajectories between groups became statistically significant by month 2, with engaged participants achieving 7.4% weight loss compared to 6.4% among nonengaged participants (absolute difference 0.95 percentage points; *P<*.001). This difference progressively increased throughout the observation period. By month 6, the absolute difference reached 3.1 percentage points (16.5% vs 13.4%; *P<*.001), and by month 8, the difference widened to 4.0 percentage points (19.3% vs 15.3%; *P<*.001).

The MMRM analysis, which controlled for demographic factors and comorbidities while accounting for the correlation structure of repeated measurements, confirmed that digital engagement was consistently associated with enhanced weight loss outcomes at all time points. This statistical approach demonstrated that the engagement effect was not attributable to baseline differences or confounding factors but represented greater weight loss outcomes associated with higher engagement.

#### Sensitivity Analyses: Weight Loss by Follow-Up Duration

Among the cohort, 10,296 (8.1%) participants reached 3 months, 5349 (4.2%) reached 6 months, 2241 (1.8%) reached 9 months, and 2653 (2.1%) reached 12 months (see Multimedia Appendix 5 for discontinuation and censoring patterns).

Linear regression analyses adjusted for baseline weight and confounders demonstrated consistent associations between engagement status and weight loss across all follow-up durations. At 3 months, nonengaged participants achieved 9.6% weight loss (95% CI 9.6-9.5) versus 10.3% (95% CI 10.5-10.2) for engaged participants (absolute difference 0.75 percentage points; *P*<.001). At 6 months, differences were 14.7% (95% CI 14.8-14.6) versus 17.6% (95% CI 17.9-17.3), respectively (absolute difference 2.9 percentage points; *P*<.001). At 9 months, nonengaged participants lost 17.8% (95% CI 18.1-17.6) compared to 21.8% (95% CI 22.4-21.2) for engaged participants (absolute difference 4.0 percentage points; *P*<.001). At 12 months, differences were 19.3% (95% CI 19.7-18.9) versus 24.2% (95% CI 25.3-23.2), respectively (absolute difference 4.9 percentage points; *P*<.001). These analyses confirmed that positive associations between engagement and weight loss were maintained regardless of the follow-up duration achieved.

#### Clinically Significant Weight Loss Thresholds

**Figure 3:**
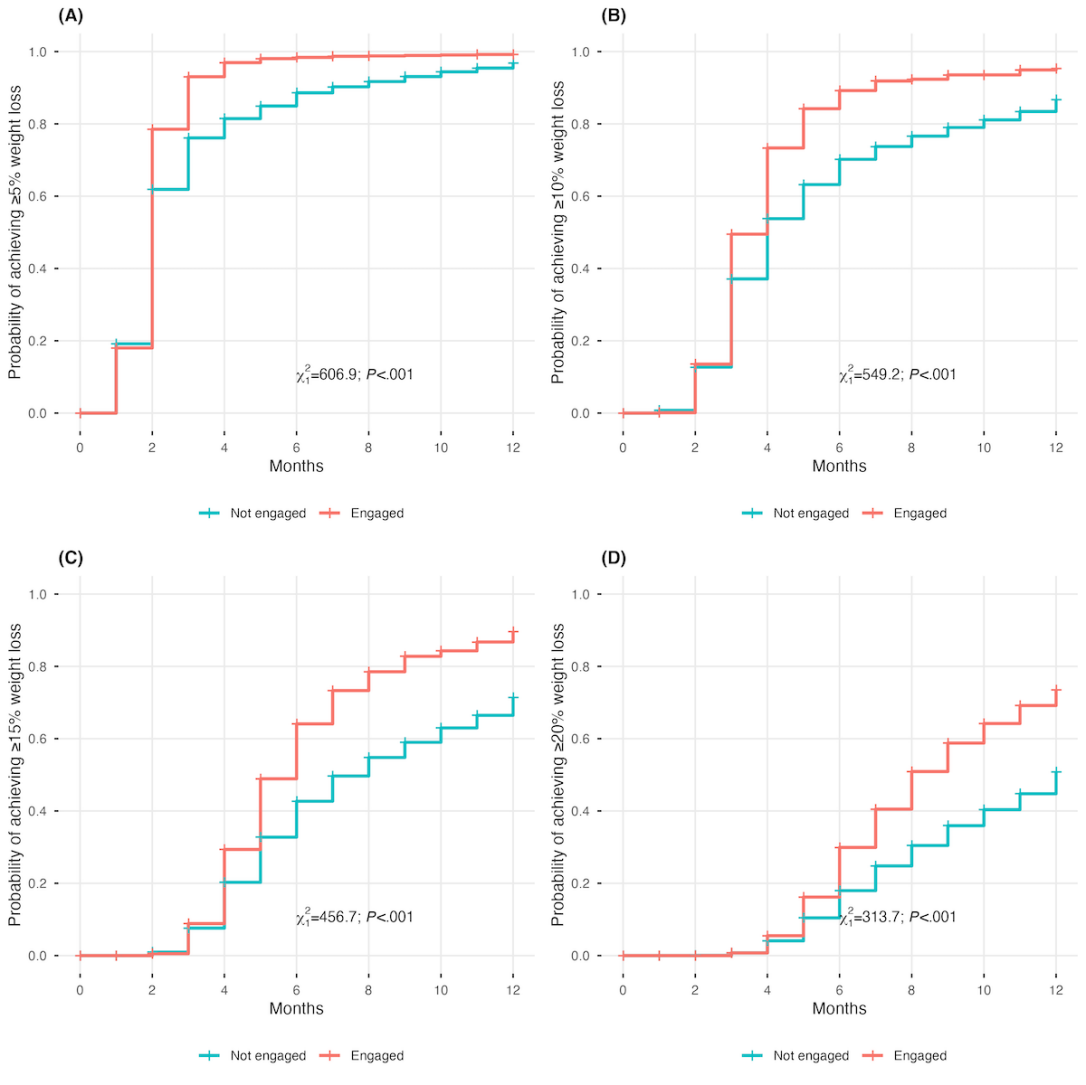
Kaplan-Meier survival curves showing the probability of achieving clinically significant weight loss thresholds over time among tirzepatide users by digital engagement status. (A) ≥5% weight loss, (B) ≥10% weight loss, (C) ≥15% weight loss, and (D) ≥20% weight loss. Red lines represent digitally engaged participants; teal lines represent nonengaged participants. Shaded areas represent 95% CIs. *P* values determined by the log-rank test. χ is derived from the chi-square test comparing engaged versus nonengaged.

Kaplan-Meier survival analysis demonstrated that tirzepatide users achieved substantial weight loss milestones rapidly, with overall cumulative probabilities reaching high levels for clinically meaningful thresholds across all participants. When stratified by engagement status, digitally engaged participants achieved these clinically significant thresholds more rapidly and at higher rates than nonengaged participants (Figure 3, log-rank *P<*.001 for all comparisons). The association between engagement and increased weight loss was evident across all thresholds but became increasingly pronounced for more substantial weight loss targets.

The cumulative probability of achieving ≥5% weight loss was substantially higher among engaged participants (5445/6746, 80.7% vs 44,329/119,807, 37%), demonstrating more than a 2-fold difference (RR 2.18; HR 1.49, 95% CI 1.45-1.53). This engagement advantage became even more pronounced for higher weight loss thresholds: ≥10% weight loss (3623/6746, 53.7% vs 26,240/119,807, 21.9%; RR 2.45; HR 1.55, 95% CI 1.50-1.60), ≥15% weight loss (2091/6746, 31% vs 14,017/119,807, 11.7%; RR 2.65; HR 1.67, 95% CI 1.60-1.75), and ≥20% weight loss (1079/6746, 16% vs 6710/119,807, 5.6%; RR 2.88; HR 1.79, 95% CI 1.68-1.91).

#### Longitudinal Participation Patterns

As expected, the number of participants with data at each time point decreased over the observation period, primarily reflecting the service’s continuous enrollment pattern rather than attrition. Given the rolling nature of the service, participants joined at various time points throughout the evaluation period, with many not yet having the opportunity to reach later time points. The mean follow-up duration was 2.6 (SD 3.0) months, with substantial variation across participants reflecting the rolling enrollment design. Among the cohort, 2653 (2.1%) participants completed 12 months of follow-up. Of these, 310 (11.7%) were maximally engaged, and 2343 (88.3%) were nonengaged. Given the nature of the open cohort study, loss to follow-up could be due to discontinuation (stopped taking medication) or censoring (was still using medication at the cohort end date; Multimedia Appendix 5).

#### Baseline Correlates of Digital Engagement

Baseline characteristics associated with digital engagement are presented in Table 3. Male participants had significantly lower odds of engagement than female participants (adjusted OR for male: 0.86, 95% CI 0.81-0.92; *P<*.001). Each 10-year increase in age was associated with 18% higher odds of engagement (adjusted OR per decade 1.18, 95% CI 1.15-1.20; *P<*.001). Every 5 kg/m^2^ increase in baseline BMI corresponded to 14% higher odds of engagement (adjusted OR 1.14, 95% CI 1.12-1.16; *P<*.001). Among comorbidities, participants with PCOS had 59% higher odds of engaging (adjusted OR 1.59, 95% CI 1.45-1.74; *P<*.001), and those with fatty liver disease had 52% higher odds (adjusted OR 1.52, 95% CI 1.32-1.76; *P<*.001). Hypercholesterolemia was associated with substantially higher engagement odds (adjusted OR 1.34, 95% CI 1.23-1.47; *P<*.001), while hypertension showed more modest associations (adjusted OR 1.16, 95% CI 1.07-1.25; *P<*.001). Notably, diabetes was associated with significantly lower odds of engagement (adjusted OR 0.83, 95% CI 0.73-0.95; *P*=.009).

**Table 3:** Baseline characteristics associated with digital engagement among tirzepatide users through multivariable logistic regression analysis^a^.

Characteristic		Adjusted OR^b^ (95% CI)	*P* value
**Demographics**			
	Female sex	Reference (—^c^)	—
	Male sex	0.86 (0.81-0.92)	<.001
	Age (per year)	1.02 (1.01-1.02)	<.001
	Age (per decade)^d^	1.18 (1.15-1.20)	<.001
	BMI (per unit)	1.03 (1.02-1.03)	<.001
	BMI (per 5 kg/m^2^)^d^	1.14 (1.12-1.16)	<.001
**Comorbidities**			
	Polycystic ovary syndrome	1.59 (1.45-1.74)	<.001
	Fatty liver disease	1.52 (1.32-1.76)	<.001
	Hypertension	1.16 (1.07-1.25)	<.001
	Hypercholesterolemia	1.34 (1.23-1.47)	<.001
	Diabetes	0.83 (0.73-0.95)	.009

^a^The dependent variable was digital engagement status (engaged vs not engaged), defined as active use of digital coaching, tracking tools, or a mobile app. All characteristics were assessed at baseline (month 0) prior to tirzepatide initiation. Multivariable logistic regression model adjusted for all variables shown.

^b^OR: odds ratio.

^c^Not applicable.

^d^Derived values to express effect sizes for clinically meaningful increments.

## Discussion

### Principal Findings

This cohort study demonstrated the notable real-world effectiveness of tirzepatide in a UK-based DWLS and highlighted an association between higher digital engagement and greater weight loss. After controlling for demographic factors and comorbidities, digitally engaged participants achieved an absolute 5.3 percentage points greater weight loss at month 12 compared to nonengaged users (*P<*.001). This engagement effect emerged gradually, with trajectories beginning to diverge around month 2 and differences progressively widening thereafter. By month 12, digitally engaged participants achieved a mean weight loss of 22.9% compared to 17.6% for nonengaged participants.

Nearly all tirzepatide users eventually achieved ≥5% weight loss regardless of engagement status, but more substantial differences were observed for the engaged group for higher weight loss thresholds. Notably, engaged participants demonstrated substantially higher cumulative probabilities of achieving ≥15% weight loss (2091/6746, 31% vs 14,017/119,807, 11.7%; HR 1.67, 95% CI 1.60-1.75) and ≥20% weight loss (1079/6746, 16% vs 6710/119,807, 5.6%; HR 1.79, 95% CI 1.68-1.91) by study end. The temporal pattern of weight loss differences is meaningful. While statistically significant differences emerged by month 2, the magnitude of difference progressively increased throughout the observation period. In other studies, a 21% weight loss end point was typically reached at week 72 as opposed to at week 52 (month 12) as evidenced in our findings [15].

The temporal differences in weight loss associated with engagement status were further highlighted in our analysis, which revealed significantly accelerated achievement of clinically meaningful weight loss thresholds. While engagement conferred substantial advantages for ≥5% weight loss achievement (5445/6746, 80.7% vs 44,329/119,807, 37%; RR 2.18; HR 1.49, 95% CI 1.45-1.53), more substantial differences emerged for more ambitious thresholds. Notably, engaged participants demonstrated substantially higher cumulative probabilities of achieving ≥10% weight loss (3623/6746, 53.7% vs 26,240/119,807, 21.9%; RR 2.45; HR 1.55, 95% CI 1.50-1.60), ≥15% weight loss (2091/6746, 31% vs 14,017/119,807, 11.7%; RR 2.65; HR 1.67, 95% CI 1.60-1.75), and ≥20% weight loss (1079/6746, 16% vs 6710/119,807, 5.6%; RR 2.88; HR 1.79, 95% CI 1.68-1.91).

Our analysis of baseline characteristics identified that female sex, older age, and higher BMI were associated with increased engagement likelihood, with male participants having 14% lower odds (OR 0.86, 95% CI 0.81-0.92), each decade of age conferring 18% higher odds (OR 1.18, 95% CI 1.15-1.20), and every 5 kg/m^2^ BMI increase associated with 14% higher odds (OR 1.14, 95% CI 1.12-1.16; all *P<*.001). Among comorbidities, PCOS (59% higher odds; OR 1.59, 95% CI 1.45-1.74), fatty liver disease (52% higher odds; OR 1.52, 95% CI 1.32-1.76), and hypercholesterolemia (34% higher odds; OR 1.34, 95% CI 1.23-1.47) were positively associated with engagement, while diabetes was associated with 17% lower engagement odds (OR 0.83, 95% CI 0.73-0.95;; *P*=.009). These findings highlight important demographic factors influencing digital health use that may inform future program design and implementation.

### Strengths and Limitations

This study has several strengths, including its large sample size, real-world setting, and comprehensive assessment of weight trajectories over time. The 126,000-participant cohort provides robust power at early time points (n=40,582 at 2 months); while 12-month completers (n=2653) match other digital studies, the real-world implementation enhances generalizability and ecological validity. Additionally, the operational definition of digital engagement captured meaningful interaction with service components while maintaining practical relevance.

While baseline differences between engaged and nonengaged participants reached statistical significance due to the large sample size (n=126,553), the absolute magnitude of these differences was modest and unlikely to be clinically meaningful. The mean age difference was less than 1 year (mean 42.3, SD 12.5 vs mean 44.9, SD 12.0 years), and the BMI difference was approximately 1 kg/m^2^ (mean 35.2, SD 6.2 vs mean 36.4, SD 6.7 kg/m^2^). These small baseline differences are insufficient to account for the substantial treatment effects observed, particularly the 7.4% absolute difference in weight loss at 2 months and the near 3-fold difference in treatment persistence. Furthermore, the consistency association between engagement and outcomes across all time points, combined with our multivariable adjustment for baseline characteristics, supports the conclusion that digital engagement genuinely enhances pharmacological efficacy rather than these findings being attributable to baseline confounding.

Several limitations warrant consideration. First, as a retrospective service evaluation, participants were not randomly assigned to engagement conditions, introducing potential selection bias, and we cannot ascertain the causality of this association. While we adjusted for observed confounders, unmeasured factors (eg, motivation and socioeconomic status) may influence both engagement and outcomes. Second, weight measurements were self-reported via home scales, potentially introducing measurement and reporting error. However, this limitation applied to both engaged and nonengaged groups, likely minimizing differential bias. We did not systematically capture concurrent participation in external nonpharmacological weight management programs; we note this as a limitation and as a priority for future data collection. Additionally, ethnicity data were not systematically collected during the study period, limiting our ability to examine potential disparities in engagement or outcomes across ethnic groups. Future implementations of DWLS should prioritize collecting comprehensive demographic data, including ethnicity and socioeconomic indicators, to ensure equity in access and outcomes and to identify populations that may benefit from targeted engagement strategies. The monthly cost for the service in this study service represents a significant financial consideration that may limit access primarily to individuals with higher socioeconomic status. This raises important considerations regarding health inequalities, as individuals who might benefit most from weight management interventions may face financial barriers to accessing digital health services that integrate pharmacotherapy with behavioral support.

Moreover, the rolling enrollment design means that participants were at different stages of their treatment journey during the evaluation period. While this reflects real-world implementation, it complicates the interpretation of longitudinal patterns. Our approach of examining both absolute outcomes at each time point and conducting subgroup analyses of participants with sufficient follow-up time helps address this limitation. Finally, our operational definition of digital engagement, while evidence-informed, represents one approach among many possible definitions. Future research should explore alternative engagement metrics and potential dose-response relationships between engagement intensity and outcomes.

### Comparison With Prior Work

The magnitude of additional weight loss associated with digital engagement was substantial in the context of standard obesity treatment. Previous research indicates that each 5% reduction in body weight confers significant cardiometabolic benefits, including improvements in glycemic control, blood pressure, and lipid profiles [16,17]. Our findings suggest that digital engagement may help individuals reach more substantial weight loss thresholds, potentially amplifying the health benefits of tirzepatide treatment. This suggests that digital engagement may have cumulative benefits, potentially by supporting medication adherence, dietary modifications, and behavioral changes that enhance long-term outcomes [18,19]. Similar patterns have been observed in other behavioral interventions for chronic disease management, where ongoing support provides incremental benefits over time [20].

The identified correlates of digital engagement offer insights for service optimization. The higher likelihood of engagement among female participants, older adults, and those with higher baseline BMI suggests that these demographic groups may find digital support particularly valuable. Conversely, younger male participants with lower BMI appear less likely to engage, potentially benefiting from targeted engagement strategies; yet, digital health engagement literature has shown that male participants benefit more than female participants [21]. Interestingly, studies have proposed that people with diabetes have a slower velocity of weight loss in both the GLP-1 [22] and GLP and GIP-1 [23] agonist exposure groups (albeit, faster velocity in tirzepatide vs semaglutide).

Furthermore, our study confirmed that diabetes status was significantly associated with a lower likelihood of digital engagement (adjusted OR 0.83, 95% CI 0.73-0.95; *P*=.009). This may reflect complex physiological and psychological mechanisms influencing weight loss velocity in people with diabetes. To address this, DWLS should consider implementing more intensive engagement and coaching interventions tailored for patients with diabetes. We propose that digital services pilot such enhanced approaches to test whether outcomes can be improved. By contrast, the positive association between comorbidities, particularly PCOS [24] and engagement, suggests that individuals with obesity-related health concerns may perceive greater value in comprehensive support services.

Several mechanisms may explain the enhanced outcomes associated with digital engagement. Health coaching provides accountability, problem-solving support, and personalized guidance that can address common barriers to weight management [25]. Regular weight tracking enhances self-monitoring, a behavioral strategy consistently associated with improved weight outcomes [8]. Additionally, digital platforms may facilitate greater medication adherence through reminders, side effect management, and addressing various concerns, particularly important for injectable medications like tirzepatide that require consistent administration [26].

Our findings align with previous digital weight management studies while revealing important distinctions. Xu et al [27] (n=153) defined engagement as any daily food tracking, identifying thresholds of 28.5%-39.4% of days for ≥3%-5% weight loss. W8Buddy used a liberal definition (any platform activity within 14 days), achieving 83.1% engagement with 0.74 kg per month additional weight loss. Our study used the strictest definition (coaching, weekly weight tracking, and app login combined), resulting in only 5.3% meeting criteria, yet demonstrating the largest effect (5.3 percentage points at 12 months) [28]. This contrast, where stricter criteria with fewer engaged participants yielding greater outcomes, suggests that multimodal engagement combining behavioral support, self-monitoring, and clinical oversight produces beneficial effects beyond single-modality interventions. Notably, partial engagement remained common (app use: 93,627/126,553, 74% and coaching: 37,224/126,553, 29.4%), and even nonengaged participants achieved substantial weight loss (17.6%), indicating that platform features provided benefit across engagement levels. These findings demonstrate that while engagement definitions substantially influence observed rates, the consistent association between digital engagement and enhanced outcomes persists across platforms and intervention designs.

The integration of digital services with pharmacotherapy represents an evolving care model addressing limitations of traditional obesity treatment approaches. By providing remote support, behavioral tools, and clinical monitoring, DWLSs may bridge critical gaps in obesity care while reducing barriers related to geography, provider availability, and stigma [29,30]. Our findings suggest that such integrated approaches may optimize the effectiveness of novel obesity pharmacotherapies.

### Implications and Future Directions

Our findings have important implications for clinical practice, health service design, and research. Clinically, practitioners should consider recommending digital support services alongside tirzepatide prescriptions, particularly for patients who may benefit from additional accountability and behavioral guidance. From a service design perspective, our results support investment in research and development for digital infrastructure that facilitates engagement, particularly during early treatment phases when engagement patterns appear to be established.

Some self-funded DWLS can exacerbate health inequalities; therefore, future research should examine the cost-effectiveness of such integrated DWLS compared to medication-only approaches and explore alternative funding models, including potential National Health Service commissioning pathways, that could improve accessibility across socioeconomic groups while maintaining service quality and clinical outcomes [31]. Additionally, qualitative studies exploring patient experiences and clinician perspectives could identify specific digital components that contribute most to engagement and outcomes. Finally, RCTs comparing different digital support intensities would address causality questions and optimize resource allocation.

### Conclusions

This study demonstrates that digital engagement was significantly associated with enhanced weight loss outcomes among individuals using tirzepatide for obesity management. By month 12, engaged participants achieved –22.9% weight loss and an absolute difference of –5.3% compared to nonengaged participants, with differences emerging early and increasing over time. These findings highlight the potential value of integrated care models combining pharmacotherapy with digital support services. As novel obesity medications continue to transform treatment possibilities, optimizing their implementation through complementary digital strategies represents a promising approach to addressing the global obesity epidemic.

## Appendix

Multimedia Appendix 1 Detailed program description.

Multimedia Appendix 2 Eligibility criteria and cohort derivation of the study.

Multimedia Appendix 3 Statistical methods and model specifications to run on R.

Multimedia Appendix 4 STROBE checklist.

Multimedia Appendix 5 Participant flow diagram showing retention, discontinuation, and censoring patterns over 12 months of follow-up.

## Abbreviations

DWLS: digital weight loss service
GIP: glucose-dependent insulinotropic polypeptide
GLP-1: glucagon-like peptide-1
HR: hazard ratio
MMRM: mixed model for repeated measures
OR: odds ratio
PCOS: polycystic ovary syndrome
RA: receptor agonist
RCT: randomized controlled trial
RR: risk ratio
STROBE: Strengthening the Reporting of Observational Studies in Epidemiology

## Data Availability

The datasets generated or analyzed during this study are not publicly available due to the nature of the clinical data collected and the consent provided by patients; the individual participant data used in this study are not publicly available. The statistical code used in the analyses can be made available to researchers upon request to the corresponding author. Requests for access to aggregated high-level anonymized data for noncommercial projects should be directed to the corresponding author, will require appropriate ethics and legal approval, and are available on reasonable request.
