# Supplementary material for: Digital Engagement Significantly Enhances Weight Loss Outcomes in Adults With Obesity Treated With Tirzepatide: Retrospective Cohort Study of a Digital Weight Loss Service
Source: J Med Internet Res. 2026 Jan 15;28:e83718. doi: 10.2196/83718 (PMC12856402; doi:10.2196/83718)
Supplement: Multimedia Appendix 1 [file jmir_v28i1e83718_app1.docx]

**Overview**

Voy is a commercial self-funded telehealth digital weight loss service integrating pharmacotherapy, clinical support, health coaching, and digital behavioral tools. The monthly cost is £209 (US $273.72), paid upfront by participants.

**Medical Management**

Participants receive branded tirzepatide (brand name: Mounjaro®) supplied via Mounjaro® KwikPen, a multi-dose prefilled pen containing four weekly doses. Tirzepatide is administered via subcutaneous injection once weekly. The starting dose is 2.5 mg, increased every 4 weeks in 2.5 mg increments to a maximum of 15 mg as tolerated. Titration is patient-led via the app, which triggers automatic clinician review of requested dose changes. Dose increases are not mandatory if not clinically indicated. New tirzepatide pens are prescribed every 28 days to ensure continuous medication supply.

**Clinical Support**

Clinical support is provided by qualified prescribing clinicians with flexible, patient-initiated scheduling via telephone, video consultations, or in-app messaging. Clinicians provide support for dose titration, side effect management, efficacy discussions, and general medical concerns. Automatic clinician review is triggered when patients request dose changes through the platform, ensuring appropriate medical oversight while maintaining convenience and accessibility.

**Health Coaching Programme**

The health coaching team comprises registered dietitians and nutritionists trained in behavioral change frameworks. The coaching approach is informed by Social Cognitive Theory, Self-Determination Theory, the Transtheoretical Model, and Theory of Planned Behaviour, utilizing motivational interviewing techniques to support sustainable behavior change. Each participant receives an initial 20 to 30 minute video or telephone consultation with a dedicated health coach assigned for the duration of treatment. Ongoing support is provided through recommended fortnightly asynchronous in-app messaging, with additional telephone or video calls arranged based on patient preference and need. The nutritional approach follows the a balanced approach minimizes common GLP-1 receptor agonist side effects. Participants also attend an initial group onboarding webinar covering medication mechanisms, lifestyle factors including diet and physical activity, and program expectations.

**Digital Platform Features**

The Voy app provides a comprehensive digital infrastructure supporting multiple aspects of the weight management programme. The platform includes a scheduling system for initial, and follow-up coaching sessions, enabling participants to book and manage appointments. Direct messaging functionality allows real-time communication with both health coaches and prescribing clinicians. An educational content library provides evidence-based materials covering nutrition fundamentals, physical activity recommendations, and behavioral strategies for weight management. The weight tracking feature enables participants to log body weight measurements with visual progress graphs showing trends over time and milestones achieved.

**Clinical Governance Framework**

The service operates under Care Quality Commission (CQC) registration, ensuring compliance with UK healthcare standards. Every participant case undergoes individual review by qualified prescribers before treatment initiation. Identity and eligibility verification includes submission of photo identification and full-body photographs for identity confirmation and BMI validation. Structured clinical questionnaires capture comprehensive medical history and screen for contraindications to tirzepatide therapy. Internal clinical audit processes provide ongoing quality assurance, whilst escalation protocols ensure prompt identification and management of safety concerns. Regular safety reviews assess adverse events, treatment outcomes, and service quality metrics. The entire clinical framework adheres to General Medical Council (GMC) and General Pharmaceutical Council (GPhC) standards for remote prescribing, ensuring patient-reported data is appropriately verified and prescribing decisions remain clinician-led with robust oversight mechanisms.

**Key Distinguishing Features**

Several features distinguish Voy from other digital weight management services. The programme integrates clinical prescribing directly with multi-modal behavioral support rather than treating these as separate service components. Coaching follows a flexible, patient-led cadence rather than fixed. The comprehensive theoretical framework underpinning behavioral interventions draws from multiple evidence-based models (see methods in main body of paper), ensuring coaching techniques are grounded in established psychological principles for sustainable behavior change.
