# Supplementary material for: Digital Engagement Significantly Enhances Weight Loss Outcomes in Adults With Obesity Treated With Tirzepatide: Retrospective Cohort Study of a Digital Weight Loss Service
Source: J Med Internet Res. 2026 Jan 15;28:e83718. doi: 10.2196/83718 (PMC12856402; doi:10.2196/83718)
Supplement: Multimedia Appendix 2 [file jmir_v28i1e83718_app2.docx]

1. **Inclusion criteria**
   - Age 18-75 years
   - BMI ≥ 30 kg/m², or ≥ 27.5 kg/m² with at least one obesity-related comorbidity
   - Access to a compatible smartphone or tablet
2. **Exclusion criteria**
   - Self-reported history of eating disorders (e.g., anorexia nervosa, bulimia nervosa)
   - Current pregnancy or actively trying to conceive
   - Known allergy/hypersensitivity to tirzepatide or excipients
   - Medical contraindications to tirzepatide, including:
     - Personal or family history of medullary thyroid carcinoma or MEN 2
     - Severe hepatic impairment
     - Renal impairment requiring dialysis
     - Uncontrolled cardiovascular disease
     - Significant gastrointestinal disorders (e.g., gastroparesis, pancreatitis)
