# Supplementary material for: Digital Engagement Significantly Enhances Weight Loss Outcomes in Adults With Obesity Treated With Tirzepatide: Retrospective Cohort Study of a Digital Weight Loss Service
Source: J Med Internet Res. 2026 Jan 15;28:e83718. doi: 10.2196/83718 (PMC12856402; doi:10.2196/83718)
Supplement: Multimedia Appendix 3 [file jmir_v28i1e83718_app3.docx]

1. **Mixed Model for Repeated Measures (MMRM)**
   - **Fixed effects:** digital engagement status, month (treated as categorical), engagement×month interaction
   - **Covariates:** sex, age, baseline BMI, and comorbidities (diabetes, hypertension, hypercholesterolemia, PCOS, fatty liver disease)
   - **Covariance structure evaluation:**
     - Compound symmetry (CS)
     - Heterogeneous autoregressive (AR1H)
     - Unstructured
   - **Model selection:** comparison of AIC/BIC, convergence diagnostics
   - **Implementation details:**
     - mmrm R package v0.3.14
     - Optimizer: L-BFGS-B, max iterations 1500
     - Denominator df via Satterthwaite’s method
   - **Missing data:** assumed missing at random; no imputation
