# Supplementary material for: Digital Engagement Significantly Enhances Weight Loss Outcomes in Adults With Obesity Treated With Tirzepatide: Retrospective Cohort Study of a Digital Weight Loss Service
Source: J Med Internet Res. 2026 Jan 15;28:e83718. doi: 10.2196/83718 (PMC12856402; doi:10.2196/83718)
Supplement: Multimedia Appendix 4 [file jmir_v28i1e83718_app4.pdf]

Supplementary File 1

STROBE Checklist JMIR Submission

Digital engagement significantly enhances weight loss outcomes in adults with obesity treated with tirzepatide: a retrospective cohort study of a digital weight loss service

STROBE Statement Checklist of items that should be included in reports of ***cohort studies***

	Item No	Recommendation
**Title and abstract**	1	(*a*) Indicate the study's design with a commonly used term in the title or the abstract
		(*b*) Provide in the abstract an informative and balanced summary of what was done and what was found
**Introduction**		
Background/rationale	2	Explain the scientific background and rationale for the investigation being reported
Objectives	3	State specific objectives, including any prespecified hypotheses
**Methods**		
Study design	4	Present key elements of study design early in the paper
Setting	5	Describe the setting, locations, and relevant dates, including periods of recruitment, exposure, follow-up, and data collection
Participants	6	(*a*) Give the eligibility criteria, and the sources and methods of selection of participants. Describe methods of follow-up
		(*b*) For matched studies, give matching criteria and number of exposed and unexposed
Variables	7	Clearly define all outcomes, exposures, predictors, potential confounders, and effect modifiers. Give diagnostic criteria, if applicable
Data sources/ measurement	8*	For each variable of interest, give sources of data and details of methods of assessment (measurement). Describe comparability of assessment methods if there is more than one group
Bias	9	Describe any efforts to address potential sources of bias

Study size	10	Explain how the study size was arrived at
Quantitative variables	11	Explain how quantitative variables were handled in the analyses. If applicable, describe which groupings were chosen and why
Statistical methods	12	(*a*) Describe all statistical methods, including those used to control for confounding
		(*b*) Describe any methods used to examine subgroups and interactions
		(*c*) Explain how missing data were addressed
		(*d*) If applicable, explain how loss to follow-up was addressed
		(*e*) Describe any sensitivity analyses
**Results**		
Participants	13*	(a) Report numbers of individuals at each stage of study—eg numbers potentially eligible, examined for eligibility, confirmed eligible, included in the study, completing follow-up, and analysed
		(b) Give reasons for non-participation at each stage
		(c) Consider use of a flow diagram
Descriptive data	14*	(a) Give characteristics of study participants (eg demographic, clinical, social) and information on exposures and potential confounders
		(b) Indicate number of participants with missing data for each variable of interest
		(c) Summarise follow-up time (eg, average and total amount)
Outcome data	15*	Report numbers of outcome events or summary measures over time
Main results	16	(*a*) Give unadjusted estimates and, if applicable, confounder-adjusted estimates and their precision (eg, 95% confidence interval). Make clear which confounders were adjusted for and why they were included
		(*b*) Report category boundaries when continuous variables were categorized
		(*c*) If relevant, consider translating estimates of relative risk into absolute risk for a meaningful time period

Other analyses	17	Report other analyses done—eg analyses of subgroups and interactions, and sensitivity analyses
**Discussion**		
Key results	18	Summarise key results with reference to study objectives
Limitations	19	Discuss limitations of the study, taking into account sources of potential bias or imprecision. Discuss both direction and magnitude of any potential bias
Interpretation	20	Give a cautious overall interpretation of results considering objectives, limitations, multiplicity of analyses, results from similar studies, and other relevant evidence
Generalisability	21	Discuss the generalisability (external validity) of the study results
**Other information**		
Funding	22	Give the source of funding and the role of the funders for the present study and, if applicable, for the original study on which the present article is based

Item No.	STROBE Recommendation	Where addressed in manuscript (section & page hints)	Pg number
**1a**	Indicate the study's design in the title or abstract.	Title includes 'a retrospective cohort study'; Abstract restates design. (Title; Abstract)	1-2
**1b**	Provide an informative, balanced abstract.	Abstract summarizes setting (UK DWLS), dates (Feb 2024–Aug 2025), N=126,553, engagement definition, MMRM/KM, key results and CIs. (Abstract)	1-2
**2**	Explain scientific background and rationale.	Introduction-Background: obesity context, tirzepatide efficacy, rationale for examining digital engagement adjuncts. (Introduction/Background)	2-3

**3**	State specific objectives/hypotheses.	Objectives: evaluate real-world tirzepatide weight loss; association of digital engagement with outcomes; predictors of engagement. (Introduction-Objectives)	2-3
**4**	Present key elements of study design early.	Methods-Study Design and Setting: retrospective open cohort of DWLS users; follow-up to 12 months. (Methods-Study Design and Setting)	3-5
**5**	Describe setting/locations/dates, including recruitment, exposure, follow-up, and data collection.	Methods-Study Design and Setting: UK digital service; period early Feb 2024–early Aug 2025; follow-up until last weight or 12 months. (Methods-Study Design and Setting)	3-5
**6a**	Eligibility criteria; sources/methods of selection; methods of follow-up.	Methods-Participants & Eligibility Criteria; Procedure explains self-enrolment and ongoing follow-up via platform. (Methods-Participants; Eligibility Criteria; Procedure)	3-5
**6b**	For matched studies, give matching criteria.	Not applicable (unmatched cohort).	n/a
**7**	Clearly define outcomes, exposures, predictors, confounders, effect modifiers; diagnostic criteria.	Methods-Primary Outcome (percent weight loss), Secondary outcomes; Engagement exposure; Predictors for logistic regression; comorbidities. (Methods-Defining Engagement and Outcome; Predictors)	5-7
**8***	For each variable, give data sources and assessment; describe comparability across groups.	Methods-Data sources/measurement embedded across sections: weights self-entered in app; engagement via app logs/coaching attendance;	5-7

		comorbidities from intake questionnaire; analyses in R 4.3.1. (Methods)	
**9**	Describe efforts to address potential bias.	Methods-Bias and Missing Data: reminders, photo options, validation checks; included all eligible initiators to reduce selection bias. (Methods-Bias and Missing Data)	5-7
**10**	Explain how the study size was arrived at.	Methods-Sample Size: power analysis for ≥10% WL threshold; minimum 118 per group stated. (Methods-Sample Size)	7
**11**	Explain handling of quantitative variables (groupings and why).	Methods-Statistical Analysis: ORs per decade age and per 5 kg/m^2 BMI; categorical thresholds for KM at 5/10/15/20%. (Methods-Predictors; Achievement of Clinically Significant Weight Loss)	5-6
**12a**	Describe all statistical methods, including confounding control.	Methods-Longitudinal Weight Loss Analysis (MMRM, covariates: age, sex, BMI, comorbidities); KM; multivariable logistic regression. (Methods-Statistics)	5-7
**12b**	Describe methods to examine subgroups/interactions.	Methods-Post-model: engagement×time interaction tested; KM stratified by engagement. (Methods-Post-Model Analyses; KM)	5-6
**12c**	Explain how missing data were addressed.	MMRM handles missing under MAR; no imputation used for primary model. (Methods-Longitudinal Weight Loss Analysis). Missing data were addressed through the MMRM approach, which utilizes all available	5-6;8

		observations without requiring complete cases. This method operates under the MAR assumption, whereby missingness may depend on observed covariates and previous measurements but not on unobserved values conditional on observed data. MMRM provides unbiased parameter estimates and maintains statistical efficiency in the presence of intermittent missing data, consistent with statistical methodology for landmark obesity trials. For time-to-event analyses, participants who remained on treatment were censored at their last prescription date or study end, with censoring appropriately incorporated into Kaplan-Meier and Cox regression models.	
**12d**	Explain how loss to follow-up was addressed.	Rolling enrolment and administrative censoring at 12 months; participation counts per timepoint reported; KM accommodates censoring. (Results-Longitudinal Participation Patterns)	5-6
**12e**	Describe any sensitivity analyses.	milestone-restricted sensitivity analysis (3/6/9/12 months) with new Ns and adjusted estimates. Sensitivity analyses were conducted and point to the Results subsection. See methods - sensitivity analysis (p7)	7 (methods) 13 (results)

		and results (p13)-Sensitivity Analyses: Weight Loss by Follow-up Duration	
**13a**	Report numbers at each stage (eligible, included, follow-up, analysed).	Results-Participant Characteristics; Figure 1 flowchart; per-timepoint Ns in Table 2. (Results-Participant Characteristics; Figure 1; Table 2)	4,8,10
**13b**	Give reasons for non-participation at each stage.	Flow and eligibility criteria in Methods; rolling enrolment context; not all reach later timepoints due to study window. (Methods-Eligibility; Results-Longitudinal Participation Patterns); censoring -Supplemantry file 2 fig1	4-10; supple file 2.fig1.
**13c**	Consider use of a flow diagram.	Figure 1 provided (cohort flow). (Results-Figure 1)	5
**14a**	Give characteristics of study participants and information on exposures and potential confounders.	Results-Participant Characteristics with Table 1 (age, sex, BMI, comorbidities) by engagement. (Results-Table 1)	8-9
**14b**	Indicate number of participants with missing data for each variable of interest.	Baseline completeness described in Data Availability; (Methods-Data Availability; Results-Table 1)	4-20
**14c**	Summarise follow-up time (average and total).	Follow-up framed as up to 12 months with rolling enrolment; counts per timepoint provided;. (Methods; Results-Longitudinal Participation Patterns)	4-5; 12-15
**15***	Report numbers of outcome events or summary measures over time.	Results-Figure 2 MMRM trajectories; Table 2 adjusted means and CIs at months 2–12. (Results-Figure 2; Table 2)	9-11

**16a**	Give unadjusted and, if applicable, adjusted estimates with precision; make clear which confounders were adjusted for and why.	Adjusted estimates (MMRM) reported with 95% CIs; covariates listed (age, sex, BMI, comorbidities). (Results-Weight Loss by Engagement Status; Methods-Statistics)	9-11
**16b**	Report category boundaries when continuous variables were categorised.	KM thresholds at ≥5/≥10/≥15/≥20% WL defined; engagement definition thresholds specified. (Methods-Achievement of Clinically Significant Weight Loss; Defining Engagement)	6-7
**16c**	Consider translating estimates of relative risk into absolute risk for a meaningful time period.	Absolute differences in pp reported in Table 2; RR/HR given for KM thresholds. (Results-KM thresholds)	14
**17**	Report other analyses (subgroups, interactions, sensitivity analyses).	Engagement×time interaction; predictors of engagement via multivariable logistic regression (Table 3). (Results-Predictors; Methods-Post-Model Analyses)	14-15
**18**	Summarise key results with reference to objectives.	Discussion-Principal Results restate objectives and main findings. (Discussion-Principal Results)	16-17
**19**	Discuss limitations, considering potential bias/imprecision.	Discussion-Strengths & Limitations: selection and measurement bias, rolling enrolment, self-reported weights, non-randomized design. (Discussion-Strengths & Limitations)	17
**20**	Give a cautious overall interpretation considering objectives, limitations, multiplicity,	Discussion-Interpretation across sections; comparison with prior work and mechanisms.	17-18

	similar studies, and other evidence.	(Discussion-Comparison with Prior Work; Implications)	
**21**	Discuss generalisability (external validity).	Discussion-Implications and Future Directions (DWLS vs traditional care; UK context). (Discussion-Implications and Future Directions)	17-20
**22**	Give the source of funding and role of funders.	Funding: 'No external funding'; conducted as routine service evaluation; ethics approval noted. (Funding; Ethical Considerations)	21-22
