# Supplementary material for: Digital Engagement Significantly Enhances Weight Loss Outcomes in Adults With Obesity Treated With Tirzepatide: Retrospective Cohort Study of a Digital Weight Loss Service
Source: J Med Internet Res. 2026 Jan 15;28:e83718. doi: 10.2196/83718 (PMC12856402; doi:10.2196/83718)
Supplement: Multimedia Appendix 5 [file jmir_v28i1e83718_app5.docx]

Commenced on DWLS with tirzepatide

126,553

Month 1

70,471

Discontinued: 14,039

Censored: 513

Month 2

55,919

Discontinued: 11,436

Censored: 625

Month 3

43,858

Discontinued: 9,730

Censored: 566

Month 4

33,562

Discontinued: 7,120

Censored: 468

Month 5

25,974

Discontinued: 5,384

Censored: 437

Month 6

20,153

Discontinued: 4,864

Censored: 485

Month 10

6,248

Month 11

4,309

Month 12

2,653

Month 7

14,804

Discontinued: 1,392

Censored: 264

Discontinued: 3,331

Censored: 360

Month 8

11,113

Discontinued: 1,691

Censored: 248

Discontinued: 2,351

Censored: 273

###

Discontinued: 1,978

Censored: 263

Month 9

8,489

Flowchart illustrating participant retention, medication discontinuation, and censoring patterns among 126,553 tirzepatide users enrolled in a UK digital weight loss service (DWLS). The cohort was stratified by digital engagement status (engaged versus non-engaged) based on baseline criteria of attendance at ≥1 coaching session AND ≥1 weekly weight log AND ≥1 app login. At each monthly timepoint, participants are categorized as: (1) continuing with data available, (2) discontinued (ceased medication and exited the service), or (3) censored (still receiving medication but reached study end date without completing full 12-month follow-up). The rolling enrollment design resulted in varying follow-up durations, with participants entering the cohort at different timepoints throughout the observation period. Numbers represent participants with weight measurement data at each specific month. Discontinuation reflects treatment cessation for any reason including adverse effects, cost, achievement of weight goals, or personal choice. Censoring reflects administrative end of observation period whilst participants remained on active treatment. This flow diagram corresponds to the sensitivity analyses reported in the main manuscript examining weight loss outcomes among sub-cohorts reaching specific follow-up milestones (3, 6, 9, and 12 months).
